# Global Epidemiology and Antimicrobial Resistance of Metallo-β-Lactamase (MBL)-Producing Acinetobacter Clinical Isolates: A Systematic Review

**DOI:** 10.3390/pathogens14060557

**Published:** 2025-06-03

**Authors:** Matthew E. Falagas, Dimitrios S. Kontogiannis, Maria Zidrou, Charalampos Filippou, Giannoula S. Tansarli

**Affiliations:** 1 Alfa Institute of Biomedical Sciences (AIBS), 9 Neapoleos Street, 151 23 Marousi, Athens, Greece; d.kontogiannis@aibs.gr (D.S.K.); m.zidrou@aibs.gr (M.Z.); 2 School of Medicine, European University Cyprus, 2404 Nicosia, Cyprus; c.filippou@euc.ac.cy; 3 Department of Medicine, Tufts University School of Medicine, Boston, MA 02111, USA; 4 Department of Laboratory Medicine and Pathology, University of Washington School of Medicine, Seattle, WA 98195, USA; tansarli@uw.edu

**Keywords:** *Acinetobacter*, β-lactamase, antimicrobial resistance, metallo-β-lactamase, New Delhi metallo-β-lactamase (NDM), imipenemase metallo-β-lactamase (IMP), Verona integron-encoded metallo-β-lactamase (VIM)

## Abstract

This systematic review assessed the global epidemiology of metallo-β-lactamase (MBL)-producing *Acinetobacter* clinical isolates and the associated antimicrobial resistance. A total of 475 relevant articles from the Cochrane Library, Google Scholar, PubMed, Scopus, and Web of Science were identified and screened as potentially eligible articles. Data from 85 articles were extracted for the analysis. Most reports on MBL-producing *Acinetobacter* clinical isolates originated from Asia [68/85 (80%) studies] and Africa [14/85 (16.5%) studies]. There were also scarce reports from Europe and America. The *bla*_VIM_ (in 31 studies), *bla*_IMP_ (in 29 studies), and *bla*_NDM_ (in 21 studies) genes were the most commonly identified genes. In 22 out of 28 (78.6%) studies with comparable data, the proportions of MBL-producing pathogens detected using phenotypic methods were numerically higher than those using genotypic methods. MBL-producing *Acinetobacter* isolates showed high resistance (up to 100%) to several antibiotic classes, including carbapenems, cephalosporins, fluoroquinolones, and monobactams. However, they showed low resistance to colistin [ranging from 0% (in six studies) to 14.3% (in one study)] and to tigecycline [0% (in three studies)]. No risk of bias assessment was conducted. The findings emphasize the global spread of MBL-producing *Acinetobacter* and the need for enhanced antimicrobial stewardship, infection control measures, and surveillance.

## 1. Introduction

Pathogens resistant to antimicrobial agents are increasing worldwide and pose a threat to public health because they cause significant mortality, morbidity, and increased healthcare costs [1]. Many of the most frequently encountered resistant isolates belong to the so-called ‘ESKAPE’ pathogens (*Enterococcus faecium*, *Staphylococcus aureus*, *Klebsiella pneumoniae*, *Acinetobacter baumannii*, *Pseudomonas aeruginosa*, and *Enterobacterales*), for which the treatment options are limited [2,3].

Among these, *Acinetobacter baumannii* infections are particularly associated with advanced antimicrobial resistance [4]. Such infections are usually healthcare associated, but may also be community acquired [5]. Moreover, patients with *Acinetobacter baumannii* infections experience high mortality [6], especially when caused by carbapenem-resistant strains [7].

The World Health Organization (WHO) has designated carbapenem-resistant *Acinetobacter baumannii* as a critical priority pathogen and has called for the development of new therapeutic options to treat these infections [8]. Furthermore, the resistance of *Acinetobacter* clinical isolates to carbapenems is increasing worldwide, driven by various mechanisms, including the production of β-lactamases [9].

Beta-lactamases inactivate β-lactam antibiotics by hydrolyzing the β-lactam ring. According to the Ambler classification, β-lactamases are divided into four molecular classes: A, B, C, and D [10,11]. Classes A, C, and D have an active serine site, whereas class B [also called metallo-β-lactamases (MBLs)] uses zinc as a cofactor [12,13]. Metal ion chelators like ethylene-diamine-tetra-acetic acid (EDTA) inhibit MBL activity [12], a property utilized in phenotypic tests to detect MBL production [14]. MBLs can hydrolyze almost all β-lactam antibiotics, including carbapenems, with the notable exception of monobactams [15,16].

Previous studies are limited in scope: some examined the regional prevalence of carbapenemase-producing *Acinetobacter* [17,18,19,20], while others focused specifically on *Acinetobacter* isolates carrying the *bla*_NDM_ gene [21]. However, a gap remains in understanding the global epidemiology of MBL-producing *Acinetobacter*. The therapeutic options for such infections are limited, as these pathogens are often resistant to most antibiotics. Potential therapeutic options include carbapenems (imipenem and meropenem), polymyxins (colistin and polymyxin B), tigecycline, and new β-lactamase inhibitor combinations (sulbactam–durlobactam and aztreonam–avibactam) [22,23,24,25]. Also, there are antibiotics in the pipeline for treating patients with these infections that could potentially be used as alternatives to the available agents, but currently they are under development in clinical trials [26]. Thus, as it is useful to evaluate the global epidemiology of MBL-producing *Acinetobacter*, this systematic review aims to address the knowledge gap by assessing the data on this clinically important issue.

## 2. Methods

### 2.1. Objectives

The objective of this review was to assess the global epidemiology of MBL-producing *Acinetobacter* clinical isolates and their resistance profiles in regard to various antimicrobial agents.

### 2.2. Eligibility Criteria

We included all the research articles reporting on *Acinetobacter* clinical isolates, with no restrictions in terms of the language, publication date, journal, region, patient demographics (adult or pediatric), or setting (inpatient or outpatient). We excluded gray literature (e.g., conference abstracts and industry reports) and any studies analyzing fewer than 5 *Acinetobacter* isolates.

We included studies that detected MBLs using genotypic methods [polymerase chain reaction (PCR)] and/or phenotypic methods [combined disk test (CDT), double-disk synergy test (DDST), or E-test]. Both CDT and DDST methods use imipenem plus EDTA disks in different settings to test for MBL production, as EDTA inhibits the action of MBLs. Thus, imipenem can act against the growth of MBL-producing strains [27,28]. For the CDT, a zone of inhibition more than 7 mm around the EDTA–imipenem-containing disk is considered a positive result for the production of MBL [27,29]. For the DDST, inhibition between the imipenem and EDTA disks, placed at a 10 mm distance, is a positive result [28,29]. For the E-test, a strip containing imipenem and EDTA is used [30]. We extracted antimicrobial susceptibility data from the studies that used antibiotic diffusion or microdilution methods for antimicrobial susceptibility testing.

### 2.3. Search Strategy

We searched five databases on 5 February 2025 (the Cochrane Library, Google Scholar, PubMed, Scopus, and Web of Science), using specific search strings (see Supplementary Table S1). Additionally, we screened the reference lists of the included studies for any further relevant articles.

### 2.4. Selection of Articles

Two investigators (DSK and MZ) conducted the searches. In Google Scholar, only the first 1000 results were accessible, and no bulk export option is available; therefore, we screened the Google Scholar results by title/abstract manually, before deduplication. All the citations from the Cochrane Library, PubMed, Scopus, and Web of Science were exported into Zotero version 7.0.11 (citation management software). These, combined with the Google Scholar selections, were deduplicated using the SR Accelerator tool. Two reviewers (DSK and MZ) independently screened the articles first based on the title and/or abstract and then by reviewing the full text. Any disagreements were resolved by consensus, during scientific meetings with a senior author (MEF).

### 2.5. Data Extraction

Two investigators (DSK and MZ) independently extracted and tabulated the key data from each study, including first author and publication year; study location (continent and country); patient population characteristics (e.g., age group, inpatient vs. outpatient); hospital/department setting; specimen type from which *Acinetobacter* isolates were obtained; identified *Acinetobacter* species; and the MBL genes tested. The main text was translated, using a web software program, if the studies were in a language other than English. For each study, we evaluated the proportion of *Acinetobacter* isolates that were MBL producers, as determined by genotypic and/or phenotypic methods. The studies were grouped based on the continent and country where the isolates were detected. If multiple data points were provided in a study regarding the isolation of MBL-producing *Acinetobacter* isolates, they were all extracted and presented separately (e.g., per isolation period). We also noted the proportion of MBL-producing isolates that were non-susceptible to various antibiotics, when such antimicrobial susceptibility data were available.

### 2.6. Adherence to the PRISMA Guidelines

This systematic review complies with the most recent “Preferred Reporting Items for Systematic Reviews and Meta-Analyses” (PRISMA) guidelines, and any omission is explicitly reported in the discussion section of this study. The study research protocol was not registered in a database. The PRISMA checklists for the abstract and full-text review are provided in Supplementary Tables S2 and S3, respectively.

## 3. Results

### Identification of Relevant Articles

**Figure 1:**
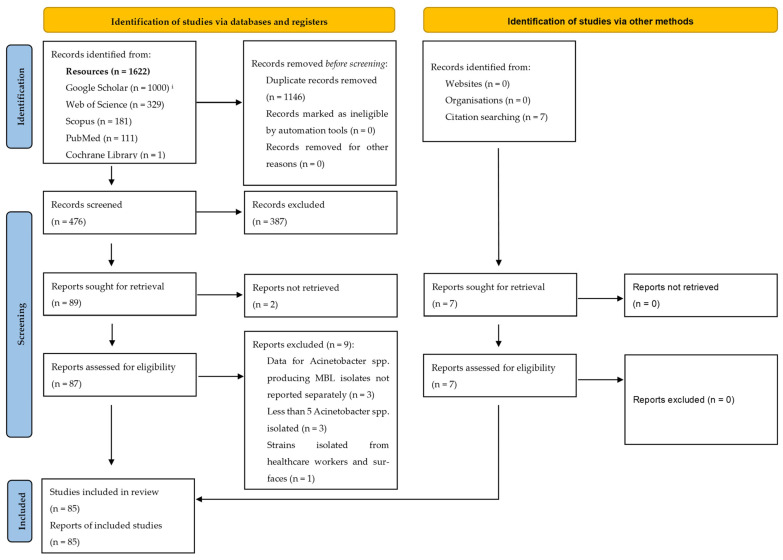
“Preferred Reporting Items for Systematic Reviews and Meta-Analyses” (PRISMA) flow diagram for identification, screening, and inclusion of articles. ^i^ Even though 16,300 results were retrieved with the Google Scholar search, only the first 1000 results could be accessed. Source: Page MJ, et al. BMJ 2021;372:n71. doi: 10.1136/bmj.n71 [116]. This work is licensed under CC BY 4.0. To view a copy of this license, visit https://creativecommons.org/licenses/by/4.0/ (accessed on 6 February 2025).

Figure 1 presents a PRISMA flow diagram on the identification, selection, and inclusion of articles included in this systematic review. In total, our searches yielded 73 articles from Google Scholar and 622 from the other sources. After removing duplicates, 475 articles remained for screening. Ultimately, 85 studies met the inclusion criteria and were included in our analysis, and nine articles were excluded after a full-text evaluation (Figure 1). Three studies did not present data specifically for MBL production in *Acinetobacter* isolates, three studies reported the isolation of less than five *Acinetobacter* pathogens, one study reported the isolation of pathogens from surfaces and healthcare workers, one study was a dissertation, and one study was a conference abstract. Sixty-eight of the included studies originated from Asia [31,32,33,34,35,36,37,38,39,40,41,42,43,44,45,46,47,48,49,50,51,52,53,54,55,56,57,58,59,60,61,62,63,64,65,66,67,68,69,70,71,72,73,74,75,76,77,78,79,80,81,82,83,84,85,86,87,88,89,90,91,92,93,94,95,96,97,98], fourteen from Africa [99,100,101,102,103,104,105,106,107,108,109,110,111,112], two from America [113,114], and one from Europe [115].

Table 1 presents the proportions of MBL-producing *Acinetobacter* isolates identified using genotypic and phenotypic methods, sorted by continent and country. In summary, 68 studies reported clinical *Acinetobacter* strains isolated from patients in countries in Asia (26 in India, 14 in Iran, 9 in Nepal, 5 in Iraq, 5 in Pakistan, 2 in Japan, 1 in Bangladesh, China, Lebanon, Malaysia, Saudi Arabia, South Korea, Taiwan), 14 in Africa (7 in Egypt, 1 in Algeria, Ghana, Libya, Morocco, South Africa, Sudan, Uganda), 2 in America (1 in Canada and the USA, 1 in Colombia), and 1 in Europe (1 in Romania). Seventy-eight studies included clinical isolates from hospitalized patients, six from both hospitalized patients and outpatients, and only one from outpatients [76]. The isolates were obtained from a variety of clinical specimens, most frequently respiratory sources (e.g., sputum and other respiratory secretions) [in 64 of 76 (84.2%) studies with available relevant data], blood [in 56/76 (73.7%)], and urine [in 50/76 (65.8%)]. Wound swabs were the next most common [in 34/76 (44.7%)], followed by cerebrospinal fluid [in 18/76 (23.7%)], pleural fluid [in 9/76 (11.8%)], burn wound samples [in 3/76 (3.9%)], and other sources.

In the 85 included studies, the most commonly identified *Acinetobacter* species were *Acinetobacter baumannii* [in 63/85 (74.1%)], followed by *Acinetobacter baumannii–calcoaceticus* complex [in 6/85 (7.1%)], and *Acinetobacter hemolyticus* [in 5/85 (5.9%)]. Other *Acinetobacter* species were also reported in 12 studies. However, 18/85 (21.2%) studies did not specify the isolated *Acinetobacter* species.

Forty-seven of the 85 studies included in our analysis (55.3%) employed genotypic methods (PCR) to detect MBL genes. In total, *bla*_VIM_ was tested in 31 studies, *bla*_IMP_ in 29, *bla*_NDM_ in 21, *bla*_SPM_ in 7, *bla*_GIM_ in 7, *bla*_SIM_ in 5, and *bla*_DIM_ in 1 study. In Africa, 10/13 (76.9%) studies tested for *bla*_NDM_, 6/13 (46.2%) for *bla*_VIM_, and 5/13 (38.5%) for *bla*_IMP_. However, in Asia, 23/31 (74.2%) studies tested for *bla*_IMP_, 22/31 (71%) for *bla*_VIM_, and 11/31 (35.5%) for *bla*_NDM_. In America, two studies tested for *bla*_VIM_, and 1/2 (50%) studies tested for *bla*_NDM_, *bla*_VIM_, and *bla*_IMP_. In Europe, the one relevant study tested for *bla*_VIM_. Only 8 of the 85 studies (9%) included in our analysis reported data on clones of *Acinetobacter baumannii* isolates.

Seventy of the 85 studies (82.4%) employed phenotypic tests (CDT, DDST, and/or E-test) for MBL detection. Notably, six studies used modified versions of these tests [52,77,79,80,109,117]. Thirty-two studies (37.6%) used both genotypic and phenotypic methods. In four of those thirty-two studies [55,59,79,112], the data were not directly comparable because the number of isolates tested using each method differed. Thus, 28 studies had directly comparable results between genotypic and phenotypic detection and were analyzed for concordance.

In 22 of the 28 studies (78.6%), phenotypic methods detected a higher proportion of MBL-producing *Acinetobacter* isolates than genotypic methods. Of those twenty-two studies, eight relied solely on the CDT for phenotypic testing [50,62,65,72,73,102,103,107], six used only the E-test [63,67,68,98,111,117], and three used only the DDST [53,57,64] method. One study used all the CDT, DDST, and E-test methods [100], and two studies used both CDT and E-test methods [51,61]. In one of the last studies, the E-test method had a lower proportion of phenotypic MBL-production detection [117/172 (68.0%)] than the genotypic method [139/172 (80.8%)] in contrast to the CDT method [144/172 (83.7%)] [51]. Also, one study used a modified CDT method, with an increase of more than 10 mm in the zone of inhibition for a positive result [80], and one used the EDTA-modified carbapenem inactivation method [117].

In 4/28 (14.3%) studies, the proportion of MBL-producing *Acinetobacter* detected was higher using genotypic than phenotypic methods. Among these four studies, two used only the DDST method [39,94], one used only the CDT method [101], and one used both CDT and DDST methods [69]. Finally, in 2/28 (7.1%) studies, the proportion of MBL-producing *Acinetobacter* detected was equal using the genotypic and phenotypic methods. One study used the CDT [31] and one used the DDST phenotypic method [99].

Table 2 presents data on the antimicrobial resistance of the studied clinical isolates. Among the 33 studies that reported antimicrobial susceptibility data for MBL-producing *Acinetobacter*, the resistance rates were as high as 100% in regard to most of the tested antibiotics, including carbapenems, cephalosporins, and fluoroquinolones. In five studies, MBL-producing pathogens showed resistance to monobactams too. Notably, in six of seven studies (85.7%) that evaluated colistin (six using antibiotic diffusion methods [34,36,55,66,71,84] and one using the agar dilution method [101]), no colistin resistance was detected among the MBL-producing isolates [34,36,55,66,71,84]. In the remaining study, colistin resistance was 14.3% (6 of 42 MBL-producing isolates) [101]. In addition, in all three studies that evaluated tigecycline, no tigecycline resistance was detected [34,44,87].

## 4. Discussion

The objective of this study was the assessment of the global epidemiology of MBL-producing *Acinetobacter* isolates and their resistance to various antimicrobial agents. Our main finding confirms that these isolates have now spread worldwide, with most reported cases coming from Asia and Africa. In most studies, MBL-producing *Acinetobacter* isolates were 100% resistant to most of the tested antibiotics, including all carbapenems. Interestingly, although MBLs do not hydrolyze monobactams, the studies that tested for aztreonam susceptibility showed high resistance to this agent. This finding implies that these isolates were co-producing other types of lactamases (such as extended-spectrum β-lactamases), thus making them resistant to aztreonam, a monobactam antibiotic.

Colistin was the only agent that retained activity against the majority of these isolates (as most studies reported 0% resistance to colistin). However, all six studies that reported 0% resistance to colistin used disk diffusion methods for antimicrobial susceptibility testing. According to the joint Clinical and Laboratory Standards Institute (CLSI)/European Committee on Antimicrobial Susceptibility Testing (EUCAST) guidelines on colistin susceptibility testing, broth and agar microdilution methods are recommended over diffusion methods [118]. Thus, colistin resistance could be underestimated in these studies, with more false-susceptible pathogens reported, and the 0% percentage of resistance to colistin could have been higher if microdilution methods had been used.

In most studies with comparable data (78.6%), MBL-producing *Acinetobacter* was more frequently detected using phenotypic methods, specifically the CDT, followed by the DDST and E-test methods. This finding indicates that the genes encoding MBLs in the isolates from these studies possibly differed from those included in the PCR assay. The fact that the CDT detected more MBL-producing isolates compared to the other phenotypic methods is in keeping with results from previous studies demonstrating that this method is more sensitive than the DDST or E-test in regard to identifying MBL-producing pathogens [119,120,121]. Moreover, in Africa, most studies tested for the presence of the *bla*_NDM_, whereas in Asia most studies tested for *bla*_IMP_ and *bla*_VIM_, highlighting the different prevalence of MBL genes between these geographical regions. Only a small proportion of studies reported data on the clones of *Acinetobacter baumannii* isolates.

Data from the included studies were heterogeneous, as patients were in different settings (ICU, other clinical departments, or outpatients) and had various infections. Also, the sources of isolation varied from study to study. These limitations made the analyses and synthesis of the data in the subgroups challenging and, thus, only a descriptive evaluation was conducted.

Antimicrobial resistance (AMR) is a growing global threat in regard to the treatment of infectious diseases. In response to rising AMR, standardized definitions for multidrug-resistant (MDR), extensively drug-resistant (XDR), and pandrug-resistant (PDR) bacteria have been adopted [122]. Briefly, MDR organisms are non-susceptible to ≥1 agent in at least three antibiotic categories, XDR organisms are resistant to all but one or two available categories, and PDR organisms are resistant to all categories [122].

Gram-negative bacteria have emerged as the most problematic causes of MDR/XDR/PDR infections from a public health perspective. In particular, *Acinetobacter baumannii*, once dismissed as a harmless colonizer, is now understood to cause severe infections. Numerous studies have demonstrated that *Acinetobacter baumannii* infections lead to considerable morbidity, prolonged hospital stays, higher healthcare costs, and attributable mortality [6,123]. Today, the need for immediate interventions and targeted research initiatives related to *Acinetobacter baumannii* infections is more urgent than ever. There is an urgent need for immediate interventions and targeted research to address *Acinetobacter baumannii* infections. Developing new antimicrobials, implementing personalized therapeutic approaches, and strengthening infection prevention and control programs are all crucial strategies to stem this crisis.

The global spread of MDR, XDR, and PDR *Acinetobacter baumannii* infections, including those caused by MBL-producing isolates, is not solely a consequence of antibiotic misuse and overuse, due to a lack of adherence to antimicrobial stewardship policies. Multiple factors, including inadequate infection control in hospitals and transmission via contaminated medical devices, also drive the spread [124]. Additionally, the genetic flexibility of *Acinetobacter baumannii* enables it to acquire and maintain resistance genes, complicating efforts to eradicate the bacteria [125]. The increasing use of invasive medical procedures, exposure to disinfectants, and heavy metals, further promote the persistence of resistant strains. Particularly concerning is the spread of MBL-producing strains, facilitated by horizontal gene transfer, plasmids, and resistance islands [125].

Extensive antibiotic resistance dramatically limits treatment options, making the management of patients with MDR *Acinetobacter* infections extremely challenging. The remaining therapeutic choices, such as polymyxins, tigecycline, and sulbactam, come with significant drawbacks, including nephrotoxicity, gastrointestinal disturbances, and limited effectiveness in certain cases [124,126,127,128]. Also, there are limited data on the clinical use of new antibiotics (cefiderocol and sulbactam–durlobactam) that may have activity against MDR *Acinetobacter* isolates. Cefiderocol was demonstrated to have considerable antimicrobial activity against Gram-negative bacterial isolates, including *Acinetobacter* baumannii [129]. Although higher mortality was observed in patients who received cefiderocol in a randomized controlled clinical trial for *Acinetobacter baumannii* infection, subsequent observational studies suggested better clinical outcomes in patients with *Acinetobacter baumannii* infection treated with this new siderophore, cephalosporin [130,131,132]. In addition, a non-inferiority randomized controlled trial comparing sulbactam–durlobactam with colistin (both combined with imipenem–cilastatin) in patients with carbapenem-resistant *Acinetobacter baumannii* infection showed promising results for this new combination of two β-lactamase inhibitors [133,134]. Notably, sulbactam may have activity against *Acinetobacter baumannii* isolates and has been used in high doses for patients with such infections [135]. This underscores the complexity of managing such infections, highlighting the urgent need for stricter surveillance, enhanced infection control programs, and the development of new therapeutic strategies [126].

Our analysis has several limitations. First, most of the included studies were from single hospitals or limited geographic areas rather than broad multicenter surveillance efforts, which may limit the generalizability of their findings. Second, there was inconsistency in the phenotypic MBL detection methods used among the studies, some used modified tests with different zone diameter cut-offs, which complicates direct comparisons of the MBL rates. Third, our analysis did not include data on some of the newest antimicrobials (e.g., recently developed β-lactam/β-lactamase inhibitor combinations), since most of the included studies were published before those agents became available. In addition, we did not perform a formal quality assessment of the included studies, sensitivity analyses, statistical methods to assess the heterogeneity of the studies, and publication bias (e.g., a tendency to report outbreaks or unusually resistant cases), which might have influenced the literature available. These factors should be kept in mind when interpreting our results.

## 5. Conclusions

The assessed data show that MBL-producing *Acinetobacter* strains that cause infections have spread globally. These isolates are associated with advanced antimicrobial resistance and pose a critical therapeutic challenge, with important consequences for global public health. These findings underscore the urgent need for a multifaceted approach, including enhanced antimicrobial stewardship, strengthened infection control measures, and sustained global surveillance, to mitigate the spread of MBL-producing *Acinetobacter* isolates.

## Figures and Tables

**Table 1:** Proportion of phenotypic and genotypic detection of MBLs in various Acinetobacter species.

Author, Year	Continent	Country	Population, Department, Hospital	Isolate Sources [n/N (%)]	Isolates (n)	Genes (n)	Genotypic Detection; Genes, n/N (%)	Phenotypic Detection; n/N (%), Method
Mesli, 2013 [99]	Africa	Algeria	Three different hospitals in western Algeria, hospital environment and patients admitted to ICU, hematology, surgery, and neurosurgery wards	Tracheal aspirate, urine, rectal swab, wound	*A. baumannii* (106) *A. radioresistens* (1) *A. nosocomialis* (2) *A. pittii* (4)	*bla*_NDM-1_	5/113 (4.4)	5/113 (4.4), DDST
Abd El-Glil, 2015 [100]	Africa	Egypt	ICU patients, Benha University, and Benha Teaching hospitals	Sputum [11/40 (17.5)], exudates [9/10 (22.5)], BAL [7/40 (17.5)], blood [6/40 (15)], urine [3/40 (7.5)]	*A. baumannii* (40)	*bla*_NDM-1_	5/40 (12.5)	26/40 (65), E-test 25/40 (62.5), CDT 22/40 (55.0), DDST
Elbrolosy, 2019 [101]	Africa	Egypt	Patients with VAP in different ICUs in Menoufia and Kasr Al Ainy University Hospitals	Tracheal aspirate [64/64 (100)]	*A. baumannii* (37) *A. calcoaceticus* (15) *A. baumannii–calcoaceticus* complex (12)	*bla*_NDM-1_	42/64 (65.6)	22/64 (34.4), CDT
El-Din, 2014 [102]	Africa	Egypt	Hospitalized patients, Tanta University Hospital	Diabetic ulcers [26/26 (100)]	*A. baumannii* (26)	*bla*_VIM_ *bla*_IMP_	Total 6/26 (23.1) *bla*_VIM_ 4/26 (15.4) *bla*_IMP_ 2/26 (7.7)	9/26 (34.6), CDT
Fattouh, 2014 [103]	Africa	Egypt	ICU patients, Microbiology Department, Sohag University	Endotracheal secretion [7/21 (33.3)], urine [6/21 (28.6)], blood [4/21 (19)], pus [4/21 (19)]	*A. baumannii* (21)	*bla*_IMP-1_ *bla*_VIM-1_	0/21 (0)	13/21 (61.9), CDT
Fouad, 2013 [104]	Africa	Egypt	ICU patients, three hospitals (6th October hospital, MUST hospital, National Cancer Institute)	Respiratory tract [24/53 (45.3)], wound [22/53 (41.5)], urine [6/53 (11.3)], blood [1/53 (1.9)]	*A. baumannii* (53)	*bla*_VIM_	1/53 (1.9)	NR
Hassan, 2021 [105]	Africa	Egypt	Hospitalized and ICU patients, Kasr Al-Aini hospital	Wound [77/206 (37.4)], respiratory secretions [56/206 (27.2), blood [37/206 (18)], urine [27/206 (13.1)], body fluid and drains [9/206 (4.4)]	*A. baumannii* (206)	*bla*_VIM_ *bla*_IMP_ *bla*_GIM_ *bla*_SPM_ *bla*_SIM-1_ *bla*_NDM-1_	Total 39/206 (18.9) *bla*_NDM-1_ 24/106 (11.7) *bla_SPM_* 13/206 (6.3) *bla*_VIM_ 1/206 (0.5) *bla*_SIM-1_ 1/206 (0.5)	NR
Wasfi, 2021 [106]	Africa	Egypt	Cancer patients at the National Cancer Institute, Giza, Egypt,	Blood [48/48 (100)]	*A. baumannii* (48)	*bla*_NDM_ *bla*_GIM_ *bla*_SPM_ *bla*_SIM_ *bla*_IMP_	31/48 (63.6)	NR
Olu-Taiwo, 2020 [107]	Africa	Ghana	Clinical isolates, patients over 50 years old, Korle-Bu Teaching Hospital	Wound [(45/87 (51.7)], urine [25/87 (28.7)], ear swabs [8/87 (9.2)], eye swabs [6/48 (6.9)] aspirates [3/48 (3.5)]	*Acinetobacter* spp. (87)	*bla*_NDM_	7/87 (8)	23/87 (26.4), CDT
Mathlouthi, 2016 [108]	Africa	Libya	Clinical isolates, Tripoli Medical Center and Burn and Plastic Surgery Hospital in Tripoli	Wound [15/36 (41.6)], catheter [3/36 (8.3)], septum [3/36 (8.3)], swab [3/36 (8.3)], urine [3/36 (8.3)], blood [2/36 (5.6)], CSF [2/36 (5.6)], chest tube [1/36 (2.8)], endotracheal tube [1/36 (2.8)], GT tube [1/36 (2.8)], mouth [1/36 (2.8)], throat [1/36 (2.8)]	*A. baumannii* (36)	*bla*_NDM-1_	7/36 (22.2)	NR
Kabbaj, 2013 [109]	Africa	Morocco	Hospitalized patients, ICU, neurosurgery ward, neurology ward, Rabat Specialty Hospital	Respiratory tract (69), urine (22), surgical site infection (5), CSF (4)	*A. baumannii* (47)	NR	NR	20/47 (24.6) ^i^
Nogbou, 2021 [110]	Africa	South Africa	Clinical isolates, teaching hospital in Pretoria	NR ^ii^	*A. baumannii* (70)	*bla*_VIM_ *bla*_IMP-5_ *bla*_NDM_ *bla*_SIM-1_	*bla*_VIM_ 60/70 (85.7) *bla*_NDM_ 41/70 (58.6) *bla*_IMP-5_ 5/70 (7.1) *bla*_SIM-1_ 2/70 (2.9)	NR
Elbadawi, 2021 [111]	Africa	Sudan	Children and adults, neonatal ICU, medicine, pediatric, and surgery wards, ICU, renal unit, Soba University Hospital	Blood (36), wound (24), urine (21), body fluids (7), catheter tips (6), sputum (6)	*A. baumannii* (36)	*bla*_NDM_	17/36 (47.2)	19/36 (52.8), E-test
Kateete, 2016 [112]	Africa	Uganda	Hospitalized patients, hospital environment, Mulago Hospital in Kampala	Hospital environment [11/40 (27.5)], tracheal aspirate [9/40 (22.5)], ear swabs [8/40 (20)], pus [4/40 (10)], blood [4/40 (10)], sputum [2/40 (5)], body fluids [1/40 (2.5)]	*A. baumannii* (40)	*bla*_VIM-1_	2/15 (40)	3/40 (7.5), DDST
Rakhi, 2019 [31]	Asia	Bangladesh	Clinical isolates, Dhaka Medical College Hospital	Blood, pleural fluid, pus, tracheal aspirate, urine, vaginal swab, wound	*A. baumannii* (4)	*bla*_NDM_	*bla*_NDM_ + *bla*_OXA-48_ 1/4 (25)	1/4 (25), CDT
Li, 2013 [32]	Asia	China	Medical and surgical wards, ICU, burn department, teaching hospital in Guangzhou	Respiratory tract [35/42 (83.3)], blood [3/42 (7.1)], wound [2/42 (4.8)], urine [1/42 (2.4)], CSF [1/42 (2.4)]	*A. baumannii* (42)	NR	NR	1/42 (2.4), E-test
Ahir, 2012 [33]	Asia	India	Hospitalized patients, tertiary care teaching hospital, Gujarat	Swab [40/78 (51.3)], urine [8/78 (10.3)], sputum [7/78 (9)], pleural fluid [7/78 (9)], pus [5/78 (6.4)], blood [67.7)], other body fluid [5/78 (6.4)] ^iii^	*A. baumannii* (40) *A. lwoffii* (20) *A. hemolyticus* (10) *A. calcoaceticus* (8)	NR	NR	78/750 (10.4), CDT and DDST
Archana Rao, 2024 [34]	Asia	India	Pediatrics, medical, surgery, ENT, and gynecology wards, Raja Rajeswari Medical College tertiary care hospital	Sputum [14/25 (56)], pus [4/25 (16)], urine [3/25 (12)], ear discharge [2/25 (8)], blood [2/25 (8)]	*Acinetobacter* spp. (25)	NR	NR	5/25 (20), CDT
Banerjee, 2015 [35]	Asia	India	Clinical isolates, Mayo Institute of Medical Sciences and Hospital, Barabanki	Endotracheal tube [17/67 (25.4)], sputum [16/67 (23.9)], pus [13/67 (19.4)], blood [9/67 (13.4)], urine [7/67 (10.4)], ascitic fluid [5/67 (7.5)]	*Acinetobacter* spp. (67)	NR	NR	16/67 (23.9), CDT
Binnani, 2018 [36]	Asia	India	Clinical isolates, Tertiary Care Institute in the North West Region of Rajasthan, India	Urine [6/21 (28.6)], sputum and respiratory tract specimens [8/21 (38.1)], blood [5/21 (23.8)], pus and other wound discharges [2/21 (9.5)]	*Acinetobacter* spp. (21)	NR	NR	8/21 (38.1), CDT
De, 2010 [37]	Asia	India	Adults, children, intensive care areas in Lokmanya Tilak Municipal Medical College and Hospital	Blood, endotracheal secretions	*Acinetobacter* spp. (25)	ΝR	NR	9/25 (36), DDST
Gautam, 2023 [38]	Asia	India	Hospitalized and outpatients, children and adults, Central Referral Hospital located in Gangtok, Sikkim	Endotracheal tube, sputum, pus, urine, blood, catheter tips, urogenital swabs	*A. baumannii* (307)	*bla*_IMP-1_ *bla*_VIM-1_	*bla*_IMP-1_ 4/100 (4) *bla*_VIM-1_ 8/100 (8)	NR
Girija, 2018 [39]	Asia	India	Patients with severe urinary tract infections	Urine [73/73 (100)]	*A. baumannii* (73)	*bla*_VIM_ *bla*_GIM_	Total 37/73 (50.7) *bla*_VIM_ 25/73 (34.2) *bla*_GIM_ 12/73 (16.4)	31/73 (42.5), DDST
Goel, 2017 [40]	Asia	India	ICU patients, teaching tertiary care hospital	Transtracheal or bronchoscopic aspirates [88/88 (100)]	*A. baumannii* (88)	NR	NR	28/37 (75.7), DDST ^iv^
Hodiwala, 2013 [41]	Asia	India	Clinical isolates	Blood, catheter tips, CSF, endotracheal secretions, pus, sputum, urine, various body fluids (synovial, ascitic, pleural)	*A. baumannii* (68)	NR	NR	9/68 (13.2), CDT and DDST
Jena, 2014 [42]	Asia	India	Outpatients, ICU, neonatal ICU, IMS and SUM Hospital in Bhubaneswar	Blood, urine, stool, pus, sputum, wound, tracheal aspiration, CSF, high vaginal swab	*Acinetobacter* spp. (66)	NR	NR	23/66 (34.8), DDST
Jethwa, 2013 [43]	Asia	India	Clinical isolates, tertiary care hospital	Swab [334/854 (39.1)], blood [278/854 (32.6)], body fluids [94/854 (11)], sputum [65/854 (7.6)], pus [39/854 (4.6)], urine [35/854 (4.1)], other [9/854 (1.1)]	*Acinetobacter* spp. (854)	NR	NR	68/854 (8), CDT
John, 2011 [44]	Asia	India	Clinical isolates, ICU patients	Urine, blood, sputum, pus, endotracheal aspirates, bronchial secretions, wound swabs, vaginal swabs	*A. baumannii* (242)	NR	NR	36/242 (14.8), DDST
Kaur, 2014 [45]	Asia	India	Clinical isolates, microbiology department	Respiratory samples, pus, blood, others, urine	*A. baumannii* (389)	NR	NR	313/389 (80.5), CDT
Kaur, 2018 [46]	Asia	India	Clinical isolates, ICU and medical wards, Microbiology Department, Adesh Institute of Medical Sciences and Research, Bathinda	Endotracheal tube secretions [34/116 (29.3)], tracheal aspirate [28/116 (24.1)], pus [29/116 (25)], urine [9/116 (7.8)], sputum [7/116 (6)], blood [6/116 (5.2)], various body fluids [3/116 (2.6)]	*A. baumannii* (116)	NR	NR	52/116 (44.8), CDT
Kumar, 2013 [47]	Asia	India	Clinical isolates, tertiary care hospital	NR ^ii^	*Acinetobacter* spp. (180)	NR	NR	43/180 (29.3), DDST
Pandya, 2016 [48]	Asia	India	Clinical isolates, medical wards including ICU, Teaching Hospital in rural Gujarat	Endotracheal secretions [26/81 (32.1)], pus [16/81 (19.8)], tracheostomy secretions [12/81 (14.8)], blood [6/81 (7.4)], sputum [6/81 (7.4)], urine [6/81 (7.4)], broncho-alveolar lavage [3/81 (3.7)], central venous catheter tip [2/81 (2.5)], ascitic fluid [1/81 (1.2)], catheter tip [1/81 (1.2)], drain [1/81 (1.2)], pleural fluid [1/81 (1.2)]	*A. baumannii* (81)	NR	NR	24/81 (29.6), CDT
Patil, 2021 [49]	Asia	India	Clinical isolates, patients with VAP, ICU, tertiary care hospital	Respiratory tract [246/246 (100)]	Total (188) *A. baumannii* (156) *A. lwoffii* (15) *A. calcoaceticus* (9) *A. hemotyticus* (5) *A. baumannii–calcoaceticus* complex (3)	NR	NR	146/188 (77.7), CDT 141/188 (75), DDST 152/188 (80.9), E-test
Rynga, 2015 [50]	Asia	India	ICU (28%), burns (15%), respiratory (15%), surgery (14%), burns ICU (10%), gynecology (9%), orthopedic (5%) wards, respiratory medicine outpatient department (2%)	Endotracheal aspirate [31/100 (31)], pus [28/100 (28)], wound [25/100 (25)], sputum [14/100 (14)], drain fluid [1/100 (1)], high vaginal swab [1/100 (1)]	*A. baumannii* (100)	*bla*_VIM_ *bla*_GIM_ *bla*_SIM_ *bla*_IMP_	Total 18/100 (18) *bla*_VIM_ 9/100 (9) *bla*_GIM_ 6/100 (6) *bla*_SIM_ 2/100 (2) *bla*_IMP_ 1/100 (1)	25/100 (25), CDT
Saikia, 2023 [51]	Asia	India	Hospitalized patients, ICU, internal medicine wards, Dibrugarh University	NR ^ii^	*A. baumannii* (172)	*bla*_NDM_ *bla*_IMP_ *bla*_VIM_	Total 139/172 (80.8) *bla*_NDM_ 121/172 (70.3) *bla*_IMP_ 88/172 (51.2) *bla*_VIM_ 42/172 (24.4)	144/172 (83.7), CDT 117/172 (68), E-test
Singla, 2013 [52]	Asia	India	Outpatients, hospitalized patients, adults and children, tertiary care hospital	Blood, BAL, CSF, endotracheal aspirates, high vaginal swabs, pus, sputum, throat swabs, urine, wound, other body fluids	Total (70) *A. baumannii* (66) *A. lwoffii* (4)	NR	NR	*A. baumannii* 38/66 (57.6) *A. lwoffii* 1/4 (25), modified CDT method ^v^
Sinha, 2013 [53]	Asia	India	Hospitalized patients, tertiary care center	Pus [52/140 (37.1)], blood [32/140 (22.6)], urine [19/140 (13.6)]	Total (140) *A. baumannii* (129) *A. lwoffii* (9) *A. hemolyticus* (2)	*bla*_IMP-1_ *bla*_VIM-1_ *bla*_VIM-2_	10/140 (7.1)	16/140 (11.4), DDST
Sugumaran, 2019 [54]	Asia	India	Hospitalized patients (81.2%), outpatients (18.8%), Mahatma Gandhi Medical College and Research Institute, Puducherry	Aspirates, central line catheter tip, ear swab, endotracheal tube, groin swab, pus, sputum, synovial fluid, tissue, urine, wound	*A. baumannii* (19)	NR	NR	10/19 (90.9), imipenem CDT 10/19 (90.9), imipenem DDST 13/19 (68.9), ceftazidime CDT 13/19 (68.9), ceftazidime DDST
Thakar, 2021 [55]	Asia	India	Hospitalized patients, outpatients, tertiary care hospital	Pus [30/72 (41.7)], respiratory tract [16/72 (22.2)], urine [16/72 (22.2)], blood [6/72 (8.3)], others [4/72 (5.6)]	*Acinetobacter* spp. (72)	*bla*_VIM_	15/15 (100)	32/72 (44.4), CDT
Tripathi, 2013 [56]	Asia	India	Clinical isolates, microbiology department	NR ^ii^	*Acinetobacter* spp. (46)	NR	NR	40/46 (87), CDT
Uma Karthika, 2009 [57]	Asia	India	ICU, acute medical care units, Pondicherry Institute of Medical Sciences tertiary care hospital	Blood, CSF, endotracheal tube, urine, wound	*A. baumannii* (36)	*bla*_IMP-1_ *bla*_VIM-2_	Total 23/54 (42.6) *bla*_IMP-1_ 23/54 (42.6) *bla*_VIM-2_ 0/54 (0)	39/54 (72.2), DDST
Vamsi, 2021 [58]	Asia	India	Hospitalized patients, SVS Medical College, Hospital in Mahabubnagar	Endotracheal tube [12/17 (70.6)], pus [2/17 (11.8)], blood [1/17 (5.9)], CSF [1/17 (5.9)], urine [1/17 (5.9)]	*Acinetobacter* spp. (23)	NR	NR	17/23 (73.9) ^vi^
Aghamiri, 2016 [59]	Asia	Iran	Hospitalized patients, 11 hospitals in Tehran	Wound [59/176 (33.5)], tracheal aspirate [34/176 (19.3)], urine [24/176 (13.6)], body fluids [20/176 (11.4)], sputum [11/176 (6.3)], catheter [10/176 (5.7)], blood [18/176 (1)]	*A. baumannii* (176)	*bla*_IMP_ *bla*_VIM_	123/176 (69.9)	165/169 (97.6), DDST
Jahantigh, 2023 [60]	Asia	Iran	Hospitalized patients, Ali Ebne Abitaleb Hospital in Zahedan, Iran	Blood (39.5), endotracheal tube (34.4), wound (20.7)	*A. baumannii* (372)	NR	NR	352/372 (94.6), CDT
Khaledi, 2019 [61]	Asia	Iran	Hospitalized patients, Kashani and Hajar Hospitals in Shahrekord	Blood, CSF, pleural effusion, trachea, urine, wound	*A. baumannii* (100)	*bla*_VIM-1_ *bla*_IMP-1_	Total 26/100 (26) *bla*_VIM-1_ 23/100 (23) *bla*_IMP-1_ 3/100 (3)	65/100 (65), E-test 59/100 (59), CDT
Maspi, 2016 [62]	Asia	Iran	Hospitalized patients, Baqiyatallah hospitals	Wound, pleural effusion, urine, blood, tracheal aspirate, BAL, sputum, ascites, abscess	*A. baumannii* (86)	*bla*_IMP_ *bla*_SPM_ *bla*_VIM_ *bla*_GIM_ *bla*_SIM_	Total 23/86 (26.7) *bla*_IMP_ 13/86 (15.1) *bla*_SPM_ 4/86 (4.7) *bla*_VIM_ 2/86 (2.3) *bla*_GIM_ 2/86 (2.3) *bla*_SIM_ 2/86 (2.3)	44/86 (51.2), CDT
Moghadam, 2016 [63]	Asia	Iran	Hospitalized patients, Nemazee and Faghihi hospitals	Sputum [35/98 (35.7)], wound (15/98 (15.3)], body fluids [13/98 (13.3)], blood [9/98 (9.2)], urine [9/98 (9.2)], endotracheal tube [8/98 (8.2)], CSF [5/98 (5.1)], BAL [2/98 (2)], axillary swab [1/98 (1)], eye swab [1/98 (1)]	*A. baumannii* (96)	*bla*_IMP_ *bla*_VIM_ *bla*_SPM_	Total 37/96 (38.5) *bla*_IMP_ 23/96 (24) *bla*_VIM_ 14/96 (14.6) *bla*_SPM_ 0/96 (0)	43/96 (44.8), E-test
Moulana, 2020 [64]	Asia	Iran	Clinical isolates, units at Babol University of Medical Sciences affiliated hospitals	Endotracheal aspirates, sputum [30/50 (60)], ulcers [12/50 (24)], urinary specimens [6/50 (12)], blood [2/50 (4)]	*A. baumannii* (50)	*bla*_VIM_	13/50 (26)	15/50 (30), DDST
Noori, 2014 [65]	Asia	Iran	Hospitalized patients, Loghman Hakim and Milad hospitals	Tracheal tube [57/108 (52.8)], urine [29/108 (26.9)], blood [8/108 (7.4)], pleural fluid [8/108 (7.4)], wound [4/108 (3.7)], other [2/108 (1.9)]	*A. baumannii* (108)	*bla*_IMP_ *bla*_SPM_	Total 3/108 (2.8) *bla*_IMP_ 3/108 (2.8) *bla*_SPM_ 0/108 (0)	86/108 (88.9), CDT
Owlia, 2012 [66]	Asia	Iran	Hospitalized patients, burn unit in Motahari Hospital, Tehran	Burns [126/126 (100)]	*A. baumannii* (126)	NR	NR	42/126 (33.3), DDST
Peymani, 2011 [67]	Asia	Iran	Hospitalized patients, tertiary care teaching hospital	Tracheal aspirate [37/100 (37)], urine [21/100 (21)], sputum [9/100 (9)], blood [7/100 (7)], catheter [6/100 (6)], bronchial washings [6/100 (6)], wound [5/100 (5)], abscess [3/100 (3)], CSF [2/100 (2)], ascites [2/100 (2)], pleural effusion [2/100 (2)]	*A. baumannii* (100)	*bla*_IMP_ *bla*_VIM_	Total 28/100 (28) *bla*_IMP_ 19/100 (19) *bla*_VIM_ 9/100 (9)	31/100 (31), E-test
Ranjbar, 2019 [68]	Asia	Iran	Patients with burns, three major hospital centers	Burns [163/163 (100)]	*A. baumannii* (163)	*bla*_IMP_ *bla*_VIM_	111/163 (68.1)	147/163 (90.2), E-test
Rezaei, 2018 [69]	Asia	Iran	ICU patients, three teaching hospitals located in Isfahan	Tracheal aspirate (68/100 (68)], CSF [10/100 (10)], wound [9/100 (9)], sputum [3/100 (3)], blood [3/100 (3)], catheters [2/100 (2)], other samples [5/100 (5)]	*A. baumannii* (100)	*bla*_IMP-1_ *bla*_VIM-1_ *bla*_VIM-2_ *bla*_IMP-2_	Total 38/100 (38) *bla*_IMP-1_ 21/100 (21) *bla*_VIM-1_ 7/100 (7) *bla*_VIM-2_ 6/100 (6) *bla*_IMP-2_ 4/100 (4)	36/100 (36), CDT 21/100 (21), DDST
Safari, 2013 [70]	Asia	Iran	Hospitalized patients, ICU, three educational hospitals in Hamadan city	Tracheal aspirate [74/100 (74)], blood [16/100 (16)], urine [5/100 (5)], sputum [4/100 (4)], wound [1/100 (1)]	*A. baumannii* (100)	NR	NR	99/100 (99), E-test
Soltani, 2018 [71]	Asia	Iran	Hospitalized patients, Nemazee tertiary care hospital	Respiratory tract [61/92 (66.3)], blood [11/92 (12)], skin [8/92 (8.7)], urine [5/92 (5.4)], body fluids [5/92 (5.4)], eyes [2/92 (2.2)]	*A. baumannii* (92)	*bla*_VIM_ *bla*_IMP_ *bla*_SPM_	76/92 (82.6)	NR
Vala, 2014 [72]	Asia	Iran	Hospitalized patients, burn unit at Shahid Motahari Hospital	Wound [28/28 (100)]	*A. baumannii* (28)	*bla*_SPM_ *bla*_IMP_ *bla*_VIM_ *bla*_DIM_ *bla*_NDM_ *bla*_GIM_	*bla*_SPM_ 1/28 (3.6)	12/28 (42.9), CDT
Al Marjani, 2013 [73]	Asia	Iraq	Clinical isolates, medical centers in Baghdad	NR ^ii^	*A. baumannii* (17)	*bla*_IMP-1_	3/17 (42.8)	7/17 (41.1), CDT
Anoar, 2014 [74]	Asia	Iraq	Clinical isolate, Burn and Plastic Surgery Hospital in Sulaimani city	Wound [44/44 (100)]	*Acinetobacter* spp. (44)	*bla*_IMP_ *bla*_VIM_	*bla*_IMP_ 19/44 (43.2) *bla*_VIM_ 5/44 (11.4)	NR
Numan, 2022 [75]	Asia	Iraq	Hospitalized patients, four hospitals in Baghdad	Sputum [35/69 (50.7)], blood [21/69 (30.4)], urine [9/69 (13)], CSF [2/69 (2.9)], wound [2/69 (2.9)]	*A. baumannii* (69)	NR	NR	51/69 (74), CDT
Radhi, 2019 [76]	Asia	Iraq	Outpatients, Hillah Teaching Hospital and Babylon Teaching Hospital for Maternity and Pediatrics	Burns [24/30 (80)], blood [4/30 (13.3)], urine [1/30 (3.3)], wound [1/30 (3.3)]	*A. baumannii* (30)	NR	NR	22/30 (73.3), E-test
Smail, 2019 [77]	Asia	Iraq	Hospitalized patients, ICU, three educational hospitals in Hamadan city	Blood, CSF, pleural fluid, pus, sputum, urine, wound	*A. baumannii* (112)	NR	NR	112/112 (100) ^vii^
Kishii, 2014 [78]	Asia	Japan	Clinical isolates, two university hospitals	Blood [123/123 (100)]	*Acinetobacter* spp. (123)	*bla*_IMP_	3/123 (2.4)	NR
Yamamoto, 2013 [79]	Asia	Japan	Clinical isolates, three university hospitals, two city hospitals in Kyoto and Shiga Prefecture	NR ^ii^	*Acinetobacter* spp. (82)	*bla*_IMP_ *bla*_VIM_ *bla*_NDM-1_	48/54 (88.9)	44/82 (53.7) ^viii^
Soudeiha, 2018 [80]	Asia	Lebanon	Hospitalized patients, Saint George Hospital University Medical Center	Respiratory tract [62/100 (62)], wound [21/100 (21)], urine [10/100 (10)], blood [4/100 (4)], catheters [3/100 (3)]	Total (100) *A. baumannii–calcoaceticus* complex (95) *A. hemolyticus* (3) *A. radioresistens* (1) *A. junii* (1)	*bla*_VIM_ *bla*_IMP_ *bla*_NDM_	0/100 (0)	Total 4/100 (4) ^ix^
Maziz, 2021 [81]	Asia	Malaysia	Clinical isolates, Selayang Hospital, Kuala Lumpur	Urine [16/50 (38)], blood [14/50 (26)], pus [7/50 (14)], skin [5/50 (10)], respiratory secretions [3/50 (6)] and sputum [3/50 (6)]	*Acinetobacter* spp. (50)	NR	NR	0/50 (0), DDST and E-test
Koirala, 2017 [82]	Asia	Nepal	Clinical isolates, B&B Hospital Kathmandu	Pus [36/109 (33)], suction tip [23/109 (21.1)], sputum [19/109 (17.4)], tracheostomy [16/109 (14.7)], catheter tip [7/109 (6.4)], central venous catheter [6/109 (5.5)], body fluids [1/109 (0.9)], urine [1/109 (0.9)]	*Acinetobacter* spp. (109)	NR	NR	48/109 (44), CDT
Kumari, 2021 [117]	Asia	Nepal	Clinical isolates, Koirala Institute of Health Sciences	Blood, pus, urine, sputum, endotracheal aspirate, exudate body fluid, central venous catheter, CSF, high vaginal swab, nasal swab, tissue, semen	Total (324) *A. baumannii–calcoaceticus* complex (167) *A. lwoffii* (83) *A. hemolyticus* (38) *A. radioresistens* (30) *A. junii* (6)	*bla*_NDM-1_	Total 33/324 (10.2) *A. baumannii–calcoaceticus* complex 28/167 (16.8) *A. junii* 1/6 (16.7) *A. hemolyticus* 2/38 (5.2) *A. lwoffii* 2/83 (2.4)	Total 70/324 (21.6) *A. baumannii–calcoaceticus* complex 56/167 (33.5) *A. lwoffii* 3/83 (3.6) *A. hemolyticus* 7/38 (18.4) *A. radioresistens* 3/30 (10.0) *A. junii* 1/6 (16.7), EDTA-modified carbapenem inactivation method
Mishra, 2012 [84]	Asia	Nepal	Clinical isolates, bacteriology laboratory at Tribhuvan University Teaching Hospital	Lower respiratory tract [60/60 (100)]	Total (62) *A. baumannii–calcoaceticus* complex (60) *A. lwoffii* (2)	NR	NR	3/62 (4.8), CDT and DDST
Pandey, 2021 [85]	Asia	Nepal	Clinical isolates, 100-bed hospital in the capital city of Nepal	Sputum [25/39 (64.1)], urine [9/39 (23.1)], pus [2/39 (5.1)], catheters and tubes [2/39 (5.1)], blood [1/39 (2.6)]	*A. baumannii* (39)	NR	NR	4/39 (10.3), CDT and E-test
Pathak, 2017 [86]	Asia	Nepal	Clinical isolates, Shahid Gangalal National Heart Centre, Kathmandu, Nepal	Urine [5/11 (45.5)], endotracheal tube [2/11 (18.2)], suction tip [2/11 (18.2)], central venous catheter tip [1/11 (9.1)], pericardial fluid [1/11 (9.1)]	*Acinetobacter* spp. (11)	NR	NR	1/11 (9.1), CDT
Sakuma, 2024 [87]	Asia	Nepal	Clinical isolates, university hospital in Nepal	Respiratory tract [28/66 (42.4)], pus [16/66 (24.2)], blood [9/66 (13.6)], wound [7/66 (10.6)], urine [3/66 (4.5)], body fluids [3/66 (4.5)]	*A. baumannii* (66)	*bla*_NDM-1_	26/66 (39.4)	NR
Shrestha, 2015 [88]	Asia	Nepal	Hospitalized patients, Tribhuvan University Teaching Hospital	Respiratory tract [60/122 (49.2)], pus [31/122 (25.4)], urine [13/122 (10.7)]	*A. baumannii* (122)	NR	NR	50/122 (41), CDT
Thapa, 2017 [89]	Asia	Nepal	Hospitalized and outpatients, Nepal Medical College, Kathmandu	Pus [21/58 (36.2)], urine [21/58 (36.2], sputum [10/58 17.2)], body fluids [6/58 (10.3)]	*A. baumannii–calcoaceticus* complex (58)	NR	NR	18/58 (31), CDT
Yadav, 2020 [90]	Asia	Nepal	Hospitalized patients, Tribhuvan University Teaching Hospital	Respiratory tract [76/161 (47.2)], pus [44/161 (27.3)], CSF [18/161 (11.1)], urine [11/161 (6.8)], blood [10/161 (6.2)], catheters [2/161 (1.2)]	*A. baumannii* (161)	ΝR	NR	109/161 (67.7), CDT
Anwar, 2016 [91]	Asia	Pakistan	Clinical isolates, children, Children’s Hospital and Institute of Child Health Lahore	Blood, body fluids, pus, sputum, tracheal secretions, urine	*A. baumannii* (66)	NR	NR	63/66 (95.5), CDT 51/66 (72.3), DDST
Hasan, 2014 [92]	Asia	Pakistan	Clinical isolates, patients with secondary or nosocomial infections from different hospitals in Pakistan	Catheters and tubes [5/19 (26.3)], tracheal aspirate [4/19 (21.1)], blood [4/19 (21.1)], pus [2/19 (10.5)], wound [2/19 (10.5)], body fluids [1/19 (5.3)]	*A. baumannii* (90)	*bla*_NDM-1_	1/90 (1.1)	NR
Irfan, 2008 [93]	Asia	Pakistan	Clinical isolates, Aga Khan University Hospital	Blood, respiratory secretions, urine, wound	*Acinetobacter* spp. (90)	NR	NR	83/90 (92.2), CDT
Rashid, 2020 [94]	Asia	Pakistan	Clinical isolates, tertiary care referral hospitals	Blood, CSF, pus, sputum, urine, vaginal swab	*A. baumannii* (12)	*bla*_NDM-1_	2/12 (16.7)	(5), DDST
Sajjad, 2019 [95]	Asia	Pakistan	Clinical isolates, Lahore General Hospital	NR ^ii^	*A. baumannii* (13) *A. junii* (1)	NR	NR	*A. baumannii* 11/13 (84.6), DDST *A. junii* 0/1 (0), DDST
Shah, 2019 [96]	Asia	Saudi Arabia	Clinical isolates, King Abdulaziz University Hospital, an 845-bed major territory care hospital in Jeddah	Tracheal aspirate [29/135 (21.5)], blood [28/135 (20.7)], wound [19/135 (14.1), urine [19/135 (14.1)], body fluids [7/135 (5.2)], catheters and tubes [9/135 (6.7)], skin [5/135 (3.7)], others [19/135 (14.1)]	*A. baumannii* (135)	*bla*_IMP_ *bla*_VIM_ *bla*_NDM_	*bla*_IMP_ 113/135 (83.7) *bla*_VIM_ 25/135 (18.5) *bla*_NDM_ 2/135 (1.5)	NR
Sung, 2015 [97]	Asia	South Korea	Clinical isolates, university hospital in Daejeon	Urine [18/21 (85.7)], sputum [2/21 (9.5)], wound [1/21 (4.8)]	*A. pittii* (21)	*bla*_IMP-1_ *bla*_NDM-1_	*bla*_IMP-1_ 19/21 (90.5) *bla*_NDM-1_ 2/21 (9.5)	NR
Lee, 2008 [98]	Asia	Taiwan	Clinical isolates, Kaohsiung Medical University Hospital	NR ^ii^	Total (185) *A. baumannii* (184) *A. hemolyticus* (1)	*bla*_VIM-2_ *bla*_VIM-3_ *bla*_VIM-11_ *bla*_IMP-8_	Total 79/185 (42.7) *A. hemolyticus* 1/1 (100) *A. baumannii* 78/184 (42.3)	Total 80/185 (43.2), E-test *A. baumannii* 79/184 (42.9) *A. hemolyticus* 1/1 (100)
Mereuţă, 2013 [115]	Europe	Romania	Clinical isolates, five university hospitals in Iasi	Urine [5/16 (31.3)], pus [5/16 (31.3)], sputum [3/16 (18.8)], tracheal aspirate [1/16 (6.3)], blood [1/16 (6.3)], CSF [1/16 (6.3)]	*A. baumannii* (16)	*bla*_VIM_	2/16 (12.5)	3/16 (18.8), E-test
Takemura, 2023 [113]	North America	Canada	Clinical specimens	NR ^ii^	*A. baumannii* (20)	*bla*_IMP_ *bla*_VIM_ *bla*_NDM_ *bla*_GIM_	*bla*_NDM_ 1/20 (5)	NR
Takemura, 2023 [113]	North America	USA	Clinical isolates, SIDERO-WT surveillance studies	NR ^ii^	*A. baumannii* (20)	*bla_IMP_* *bla_VIM_* *bla_NDM_* *bla_GIM_*	*bla*_NDM_ 2/20 (10)	NR
Hernández-Gómez, 2014 [114]	South America	Colombia	Adult, pediatric, and neonatal ICU patients, 23 clinics and hospitals	Blood, urine, respiratory tract, other	*A. baumannii* (241)	*bla*_VIM_	0/241 (0)	NR

Abbreviations: MBL, metallo-β-lactamase; DDST, double-disk synergy test; BAL, bronchoalveolar lavage; CDT combined disk test; VAP, ventilator-associated pneumonia; CSF, cerebrospinal fluid. Notes: ^i^ >17 mm zone of inhibition positive for MBL production; ^ii^ not available data for the specific sources of isolation; ^iii^ information available only for MBL-producing strains; ^iv^ 37 meropenem-resistant isolates were tested for MBL production; ^v^ 30 μg ceftazidime, 10 μg imipenem, 0.5 M EDTA, ceftazidime with ≥5 mm zone of inhibition, and/or meropenem with ≥7 mm zone of inhibition positive for MBL production; ^vi^ does not specify which test produced the specific results for *Acinetobacter* spp. (CDT, DDST, or E-test); ^vii^ modified CDT method: meropenem, imipenem, 0.35 M EDTA, ≥2 mm zone of inhibition considered positive for MBL production; ^viii^ modified CDT method: two disks 30 μg ceftazidime, one disk 3 mg sodium mercaptoacetic acid, ≥5 mm zone of inhibition considered positive for MBL production; ^ix^ modified CDT method: meropenem and imipenem, 5 mM EDTA, ≥10 mm zone of inhibition considered positive for MBL production.

**Table 2:** Proportion of the studied resistant MBL-producing Acinetobacter clinical isolates to various antimicrobial agents.

Author, Year	Continent	Country	Isolates (n)	Resistance n/N (%) to Antimicrobial Agent(s) ^i^
Abd El-Glil, 2015 [100]	Africa	Egypt	*A. baumannii* (40)	5/5 (100) imipenem, meropenem, piperacillin, cefotaxime, ceftazidime, cefepime, aztreonam, ciprofloxacin 4/5 (80) amikacin 3/5 (60) gentamicin
Elbrolosy, 2019 [101]	Africa	Egypt	*A. baumannii* (37), *A. calcoaceticus* (15), *A. baumannii–calcoaceticus* complex (12)	42/42 (100) imipenem, meropenem, cefotaxime, ceftriaxone, ceftazidime, cefepime, cotrimoxazole, piperacillin–tazobactam, tetracycline, aztreonam, ciprofloxacin, amikacin 6/42 (14.3) colistin
Olu-Taiwo, 2020 [107]	Africa	Ghana	*Acinetobacter* spp. (87)	23/23 (100) ampicillin, cefotaxime, ceftazidime, cefuroxime, meropenem 22/23 (95.7) amoxicillin–clavulanate, levofloxacin 21/23 (91.3) gentamicin 20/23 (87) ciprofloxacin 17/23 (73.9) cotrimoxazole 14/23 (60.9) nitrofurantoin 8/23 (34.8) amikacin
Kateete, 2016 [112]	Africa	Uganda	*A. baumannii* (40)	3/3 (100) ciprofloxacin, imipenem, meropenem, piperacillin–tazobactam 2/3 (66.7) gentamicin, ceftazidime, aztreonam, amikacin
Archana Rao, 2024 [34]	Asia	India	*Acinetobacter* spp. (25)	0/5 (0) colistin, tigecycline 5/5 imipenem and/or meropenem
Binnani, 2018 [36]	Asia	India	*Acinetobacter* spp. (21)	8/8 (100) ceftazidime, doxycycline, imipenem, meropenem, nitrofurantoin 7/8 (87.5) ceftriaxone, ciprofloxacin 5/8 (62.5) amikacin 0/8 (0) colistin, polymyxin B
De, 2010 [37]	Asia	India	*Acinetobacter* spp. (25)	9/9 (100) imipenem, gentamicin, amikacin, netilmicin, amoxicillin–clavulanic acid, cefotaxime, ceftriaxone, ceftazidime, cefepime, ciprofloxacin, ofloxacin, piperacillin, piperacillin–tazobactam
John, 2011 [44]	Asia	India	*A. baumannii* (242)	36/36 (100) ciprofloxacin, piperacillin, gentamicin, ceftazidime 0/36 (0) tigecycline
Kaur, 2014 [45]	Asia	India	Total (1017), *A. baumannii* (964), *A. lwoffii* (48), *A. hemolyticus* (5)	*A. baumannii*: ^ii^ 313/313 (100) imipenem 309/313 (98.7) ceftazidime 307/313 (98.1) ciprofloxacin 305/313 (97.4) cotrimoxazole 304/313 (97.1) cefepime 295/313 (94.2) gentamicin 285/313 (91.1) piperacillin 273/313 (87.2) amikacin 209/313 (66.8) netilmicin 179/313 (57.2) piperacillin–tazobactam
Pandya, 2016 [48]	Asia	India	*A. baumannii* (81)	24/24 (100) ampicillin–sulbactam, ceftazidime, ciprofloxacin, gentamicin, ticarcillin–clavulanic acid, ceftriaxone, piperacillin
Patil, 2021 [49]	Asia	India	Total (188), *A. baumannii* (156), *A. lwoffii* (15), *A. calcoaceticus* (9), *A. hemotyticus* (5), *A. baumannii–calcoaceticus* complex (3)	164/164 (100) piperacillin, piperacillin–tazobactam, ciprofloxacin, ceftazidime, cefepime, imipenem, meropenem 162/164 (98.8) ceftriaxone 152/164 (92.7) tetracycline 147/164 (89.6) doxycycline 143/164 (87.2) gentamicin 137/164 (83.5) amikacin 131/164 (79.9) cotrimoxazole
Singla, 2013 [52]	Asia	India	Total (70), *A. baumannii* (66), *A. lwoffii* (4)	39/39 (100) cefepime, ceftriaxone, imipenem 38/39 (97.4) amoxicillin–clavulanic acid, ticarcillin–clavulanic acid 37/39 (94.8) cefotaxime, ceftazidime 35/39 (89.7) cotrimoxazole 34/39 (87.1) gentamicin 33/39 (84.6) doxycycline 30/39 (76.9) amikacin, ciprofloxacin 27/39 (69.2) netilmicin 25/39 (64.1) piperacillin–tazobactam
Thakar, 2021 [55]	Asia	India	*Acinetobacter* spp. (72)	32/32 (100) ampicillin–sulbactam, carbapenem, third and fourth generation cephalosporins 29/32 (90.6) fluoroquinolones 28/32 (87.5) amikacin 0/32 (0) colistin
Khaledi, 2019 [61]	Asia	Iran	*A. baumannii* (100)	65/65 (100) imipenem, meropenem
Owlia, 2012 [66]	Asia	Iran	*A. baumannii* (126)	42/42 (100) cefotaxime, ceftazidime, piperacillin–tazobactam, aztreonam, ciprofloxacin, amikacin, imipenem, piperacillin, ticarcillin, ticarcillin–clavulanic acid, kanamycin 12/42 (28.6) tobramycin, gentamicin 0/42 (0) colistin
Soltani, 2018 [71]	Asia	Iran	*A. baumannii* (92)	76/76 (100) cotrimoxazole, ciprofloxacin, imipenem, meropenem, ticarcillin–clavulanic acid, levofloxacin 0/76 (0) colistin, polymyxin B
Al-Marjani, 2013 [73]	Asia	Iraq	*A. baumannii* (17)	7/7 (100) cefoxitin, ceftriaxone, amoxicillin–clavulanic acid, cefepime, aztreonam
Kishii, 2014 [78]	Asia	Japan	*Acinetobacter* spp. (123)	3/3 (100) imipenem, meropenem 2/3 (66.7) levofloxacin, ciprofloxacin 1/3 (33.3) amikacin
Mishra, 2012 [84]	Asia	Nepal	*A. baumannii–calcoaceticus* complex (60)	2/3 (66.7) imipenem, meropenem 0/3 (0) colistin, polymyxin B
Pandey, 2021 [85]	Asia	Nepal	*A. baumannii* (39)	4/4 (100) imipenem 0/4 (0) polymyxin B
Sakuma, 2024 [87]	Asia	Nepal	*A. baumannii* (66)	26/26 (100) imipenem, meropenem, ceftazidime, cefotaxime, amikacin, ciprofloxacin 0/26 (0) tigecycline
Irfan, 2008 [93]	Asia	Pakistan	*Acinetobacter* spp. (90)	83/83 (100) imipenem

Notes: ^i^ defined as the proportion of resistant MBL-producing *Acinetobacter* isolates to specific antimicrobial agents among all the studied MBL-producing *Acinetobacter* isolates; ^ii^ only 313 Acinetobacter baumannii isolates were tested for antimicrobial susceptibility.

## Data Availability

The data used in the conduction of this study are available upon request.

## Supplementary Materials

The following supporting information can be downloaded at: https://www.mdpi.com/article/10.3390/pathogens14060557/s1, Table S1: Search strings for each resource; Table S2: “Preferred Reporting Items for Systematic Reviews and Meta-Analyses” (PRISMA) checklist for the abstract; Table S3: “Preferred Reporting Items for Systematic Reviews and Meta-Analyses” (PRISMA) checklist for the full-text review.

## Abbreviations

AMR	Antimicrobial resistance
CDT	Combined disk test
CLSI	Clinical and Laboratory Standards Institute
DDST	Double-disk synergy test
EDTA	Ethylene-diamine-tetra-acetic acid
EUCAST	European Committee on Antimicrobial Susceptibility Testing
MBL	Metallo-β-lactamase
MDR	Multidrug resistant
PCR	Polymerase chain reaction
PDR	Pandrug resistant
PRISMA	Preferred Reporting Items for Systematic Reviews and Meta-Analyses
WHO	World Health Organization
XDR	Extensively drug resistant
