# Supplementary material for: Global Epidemiology and Antimicrobial Resistance of Metallo-β-Lactamase (MBL)-Producing Acinetobacter Clinical Isolates: A Systematic Review
Source: Pathogens. 2025 Jun 3;14(6):557. doi: 10.3390/pathogens14060557 (PMC12196140; doi:10.3390/pathogens14060557)
Supplement: Supplementary file 1 [file pathogens-14-00557-s001.zip › Supplementary table S1.pdf]

Supplementary Table S1. Search strings for each resource[a].

Platform	Search string	Coverage (years)	Results
Google Scholar	(metallo-beta-lactamase OR MBL) AND (acinetobacter) AND (global OR international OR worldwide) AND (epidemiology OR prevalence OR dissemination)	NA	16,300[b]
Web of Science	(metallo-beta-lactamase OR MBL) AND (acinetobacter) AND (global OR international OR worldwide) AND (epidemiology OR prevalence OR dissemination)	1900 – 2025	329
Scopus	(metallo-beta-lactamase OR MBL) AND (acinetobacter) AND (global OR international OR worldwide) AND (epidemiology OR prevalence OR dissemination)	1788 – 2025	181
PubMed	(metallo beta lactamase) AND (acinetobacter) AND (global OR international OR worldwide) AND (epidemiology OR prevalence)	1946 – 2025	111
Cochrane Library	(metallo-beta-lactamase OR MBL) AND (acinetobacter) AND (global OR international OR worldwide) AND (epidemiology OR prevalence OR dissemination)	1993 – 2025	1

[a] The search was performed on 5 February 2025

[b] Only the first 1,000 results could be accessed in Google Scholar

Abbreviations: NA: not available
